# *Trans*-Resveratrol Supplementation and Endothelial Function during the Fasting and Postprandial Phase: A Randomized Placebo-Controlled Trial in Overweight and Slightly Obese Participants

**DOI:** 10.3390/nu9060596

**Published:** 2017-06-12

**Authors:** Sanne M. van der Made, Jogchum Plat, Ronald P. Mensink

**Affiliations:** 1 Department of Human Biology, NUTRIM School of Nutrition and Translational Research in Metabolism, Maastricht University Medical Center, 6200 MD Maastricht, The Netherlands; sanne.vandermade@maastrichtuniversity.nl (S.M.v.d.M.); j.plat@maastrichtuniversity.nl (J.P.); 2 Top Institute Food and Nutrition (TIFN), 6700 AN Wageningen, The Netherlands

**Keywords:** flow-mediated vasodilation, vascular function, *trans*-resveratrol, postprandial, humans

## Abstract

Studies on the effects of the long-term intake of *trans*-resveratrol on vascular function are conflicting. In addition, postprandial effects of long-term *trans*-resveratrol intake on endothelial function are not known. We therefore supplemented 45 overweight and slightly obese volunteers (25 men and 20 women) with a mean (±SD) age of 61 ± 7 years and body mass index of 28.3 ± 3.2 kg/m^2^ in random order *trans*-resveratrol (2 × 75 mg/day) or placebo capsules for 4 weeks, separated by a washout period of at least 4 weeks. At the end of each intervention period, brachial artery flow-mediated vasodilation (FMD) was measured before and after meal consumption. Plasma biomarkers for endothelial function, inflammation, and glucose and lipid metabolism were also determined. Compared with the placebo, *trans*-resveratrol did not affect fasting FMD (2.9 ± 1.4% vs. 3.0 ± 1.9%; *p* = 0.69). After the postprandial test, changes in FMD (−0.7 ± 2.3% vs. 0.2 ± 2.6%; *p* = 0.13) were also not significantly different. Postprandial changes in biomarkers were also comparable. In conclusion, for overweight and slightly obese volunteers, a daily intake of 150 mg of *trans*-resveratrol for 4 weeks does not change plasma biomarkers of endothelial function or inflammation in the fasting state or postprandial phase.

## 1. Introduction

Resveratrol, a plant polyphenolic compound, found for example in the skin of black grapes, may reduce the risk of coronary heart diseases and diabetes [1]. However, earlier we have reported that a daily supplement of 150 mg of *trans*-resveratrol, the active isomer of resveratrol, for 4 weeks did not improve fasting metabolic risk markers in overweight and slightly obese men and women [2]. This result is in accordance with several other human studies also not observing effects of resveratrol supplementation on fasting lipid and glucose concentrations [3]. Further, a study on type 2 diabetic individuals did not find an effect on postprandial glucose concentrations [4]. Nevertheless, *trans*-resveratrol might affect other parameters related to cardiovascular health, such as vascular endothelial dysfunction.

In vitro evidence for beneficial effects of resveratrol on endothelial function was provided by Wallerath and colleagues [5], who demonstrated that in human umbilical vein endothelial cells (HUVECs), resveratrol up-regulated endothelial nitrogen oxide synthase (eNOS) mRNA and protein expressions, as well as nitrogen oxide (NO) production, in a time- and concentration-dependent manner. NO plays an essential role in the maintenance of vascular tone and reactivity. A later study with HUVECs using more physiologically relevant concentrations suggested that other factors also contribute to the positive effects of resveratrol on endothelial function, such as sirtuin 1 (SIRT1) up-regulation and cyclooxygenase 1 (COX-1) inhibition [6].

In humans, endothelial function can be assessed non-invasively by flow-mediated dilation (FMD) [7]. Wong et al. [8] have reported that the acute intake of 30, 90 or 270 mg of *trans*-resveratrol dose-dependently improved FMD in overweight and obese adults with elevated blood pressure. Obese, but otherwise healthy adults, also showed an improvement in FMD after a daily intake of 75 mg of *trans*-resveratrol for 6 weeks [9]. However, in another study, no effect on FMD was observed in 40 post-infarction patients after the intake of 10 mg of *trans*-resveratrol daily for 3 months [10]. In addition, the effects of long-term *trans*-resveratrol intake on endothelial function during the postprandial phase have not been studied. A high-fat dietary challenge may negatively influence FMD, possibly through increasing the inflammatory response and oxidative stress, which may reduce endothelial function [11,12,13,14]. Such a dietary challenge can therefore be used to evaluate subtle changes in endothelial function following long-term *trans*-resveratrol intake. Therefore, the main objective of the present study was to examine the effects of a daily intake of 150 mg *trans*-resveratrol supplementation for 4 weeks on endothelial function in the fasting state and postprandial phase in overweight and slightly obese participants.

## 2. Materials and Methods

### 2.1. Participants and Study Design

Overweight (body mass index, BMI, kg/m^2^: 25–30) and obese (BMI: 30–35), healthy participants aged between 45 and 70 years (mean ± SD: 61 ± 7 years) participated in a double-blind, randomized, placebo-controlled, cross-over study. Inclusion and exclusion criteria have been described previously [2]. Briefly, all participants were apparently healthy and did not suffer from disturbances in lipid or glucose metabolism, nor cardiovascular diseases. The inclusion criterion for serum HDL cholesterol concentrations was <1.21 mmol/L for men and <1.53 mmol/L for women, as the primary aim of the study was to examine the effects of *trans*-resveratrol supplementation on serum apoA-I concentrations. These cut-off values were the mean values found in the PROCAM study [15]. Participants were informed about the aim of the study and nature and risk of the experimental procedures before written informed consent was obtained. This study was conducted according to the guidelines laid down in the Declaration of Helsinki as revised in 1983, approved by the Ethics Committee of the Maastricht University Medical Centre, and registered on 17 January 2011, at ClinicalTrials.gov with the number NCT01364961. One hundred and eight participants were screened for eligibility, of which 58 did not meet the inclusion criteria. Thus, 50 participants started the study.

Subjects started the 4-week intervention with either *trans*-resveratrol (2 × 75 mg/day; resVida, 99.9% *trans*-resveratrol) or placebo (2 × 58 mg/day cellulose) capsules. At the start, subjects received more capsules than they needed for the entire period to account for unforeseen losses. They were instructed to take one capsule at lunch and another capsule at dinner. After a washout period of at least 4 weeks, regimens were crossed over. All capsules were provided by DSM Nutritional Products Ltd. (Kaiseraugust, Switzerland).

Subjects were weighed at each visit (days 0, 25 and 28 of each period). On day 28, a postprandial test started in the morning after a 12 h overnight fast. Participants were asked to refrain from any strenuous physical exercise and to not consume alcohol on the day before testing. Before and 240 min after a meal intake, FMD and arterial stiffness were measured. After the fasting vascular measurements, an intravenous cannula (Becton, Dickinson and Company, Franklin Lanes, NY, USA) was inserted into an antecubital vein, and a fasting blood sample (T0) was collected. Participants were then asked to consume a test meal within 10 min. Subsequent blood samples were drawn 15 (T15), 30 (T30), 45 (T45), 60 (T60), 90 (T90), 120 (T120), 180 (T180) and 240 (T240) min after meal consumption. Participants were allowed to drink water at their convenience throughout the postprandial test.

### 2.2. Test Meal

During each postprandial test, participants received two muffins and 300 mL of skim milk (0% vet Melkdrank, Friesland-Campina, Friesland, The Netherlands). The meal provided 4598 kJ, 26.5 g of protein, 121 g of carbohydrates and 56.6 g of fat (Table S1; protein/carbohydrate/fat: 9.6% of energy/44.6% of energy/46.6% of energy). One batch of muffins was prepared for the entire study and stored at −20 °C until consumption.

### 2.3. Flow-Mediated Dilation and Arterial Stiffness

FMD of the brachial artery, a measure of endothelium-dependent vascular reactivity, was measured after at least 15 min of supine rest, as described [7]. The same trained and blinded ultrasonographer performed all measurements. For 29 participants, all FMD measurements were made with a high-resolution ultrasound system using a 3–11 MHz wide-band linear array transducer (Philips Sonos 5500 system; Philips Ultrasound, Andover, MA, USA). For 14 participants, all FMD measurements were performed with a 5–10 MHz linear array probe (Picus I, Esaote Biomedica, Genoa, Italy). Images of the brachial artery were taken longitudinally, 2–10 cm above the antecubital fossa in combined Doppler/brightness mode (B-mode). A probe holder was used to secure the position of the transducer during the measurement. After a 3-min baseline measurement, a peripherally hypoxic state was induced by inflation of a pneumatic tourniquet (Hokanson TD312 automatic cuff inflator, D.E. Hokanson, Inc., Bellevue, WA, USA) placed around the forearm, inflated to a pressure of at least 50 mmHg above systolic pressure, with a minimal pressure of 200 mmHg. After 5 min, the cuff was deflated, resulting in reactive hyperemia, which was recorded for another 5 min. Images were continuously recorded on DVD during the entire 13-min measurement protocol. Acquired images were analyzed using an in-house developed, semi-automated image-analysis algorithm (Prof. A.P. Hoeks, Department of Biomedical Engineering, Maastricht University Medical Center, Maastricht, The Netherlands). FMD responses were expressed as the percentage increase in brachial artery diameter from baseline to maximal dilation post-occlusion.

Arterial stiffness was assessed by carotid-femoral pulse wave velocity (PWV_cf_) measurements (SphygmoCor CPV System, AtCor Medical Pty. Ltd., West Ryde, Australia), as described [16]. Measurements were made after at least 30 min of supine rest. An automated sphygmomanometer (Mobilograph NG, IEM GmbH, Stolberg, Germany) was used to measure supine blood pressure. Peripheral and central augmentation indexes (Aix_p_ and Aix_c_, respectively) were measured at the radial site by tonometry (SphygmoCor CPV System, AtCor Medical Pty. Ltd., West Ryde, Australia), as described [17].

FMD and arterial stiffness measurements were performed in a quiet, temperature-controlled, darkened room at baseline and 4 h postprandially (day 28 of each 4-week period).

### 2.4. Blood Analyses

Blood was sampled in ethylene diamine tetraacetic acid (EDTA)-containing, sodium fluoride (NaF)-containing, and serum separator tubes (Becton, Dickinson and Company, Franklin Lanes, NY, USA). Blood drawn into serum separator tubes was allowed to clot for at least 30 min at 21 °C, and was followed by centrifugation at 1300× *g* for 15 min at 21 °C. EDTA- and NaF-containing tubes were immediately placed on ice after blood drawing. Tubes were centrifuged at 1300× *g* for 15 min at 4 °C. Plasma and serum aliquots were directly snap-frozen in liquid nitrogen and stored at −80 °C until analysis.

Plasma glucose concentrations were measured in NaF plasma for T0, T15, T30, T45, T60, T90, T120 and T240 (Horiba ABX, Montpellier, France). Concentrations of triacylglycerol, corrected for free glycerol, were determined in serum samples from T0, T60, T120, T180 and T240 (GPO Trinder; Sigma Diagnostics, Santa Clara, CA, USA). Insulin concentrations were measured in EDTA plasma for T0, 15, 30, 45, 60, 90, 120, 180 and 240 (RIA; Millipore, Billerica, MA, USA). Interleukin 6 (IL-6), tumor necrosis factor alpha (TNFα), soluble endothelial selectin (sE-selectin), soluble thrombomodulin (sTM), soluble platelet selectin (sP-selectin), soluble intercellular adhesion molecule-1 (sICAM-3), soluble intercellular adhesion molecule-1 (sICAM-1), and soluble vascular adhesion molecule-1 (sVCAM-1) were measured in EDTA plasma for T0, 120 and 240 by commercially available Multi Spot ELISA kits (Meso Scale Discovery, Rockville, MD, USA).

### 2.5. Statistical Analyses

Data are presented as means ± standard deviation (SD), unless otherwise indicated. Results obtained during the fasting state at the end of the *trans*-resveratrol and placebo periods, as well as changes within a group, were compared using a paired *t*-test. For plasma glucose, plasma insulin and serum triacylglycerol, the area under the curve (AUC) and the incremental area under the curve (iAUC) were calculated using the trapezoidal rule [18]. Maximal changes in concentrations were calculated by subtracting baseline values from maximal or minimal values. Differences in AUC, iAUC and maximal changes between the test periods were tested for significance by a paired *t*-test.

Changes over time for plasma glucose, plasma insulin, serum triacylglycerol, plasma sE-selectin, plasma sICAM-1, plasma sICAM-3, plasma sP-selectin, plasma sTM, plasma sVCAM-1, plasma IL-6, and plasma TNFα were tested by linear mixed models, with “meal” and “time” as fixed factors and “meal × time” as the interaction term. If the interaction term reached statistical significance, differences in responses between the meals were compared at each individual time point using a Bonferroni correction. If the interaction term was not statistically significant, it was omitted from the model. If, in that model, the factor time was significant, post hoc tests with Bonferroni correction were carried out to compare concentrations at the different time points to baseline concentrations.

For FMD and PWV_cf_, changes between fasting and postprandial values were calculated. Differences between the two test periods were evaluated for fasting measurements and postprandial changes by a student’s paired *t*-test. Post hoc exploratory data analyses were performed to examine whether the results depended on sex, degree of obesity, or the use of prescribed medication. Carry-over effects of these responses, which were absent, were analyzed as described [19]. Results were considered to be statistically significant if *p* ≤ 0.05. Statistical analyses were performed using SPSS 19.0 for Mac OS X (SPSS Inc., Chicago, IL, USA).

## 3. Results

### 3.1. Study Participants

The flow of participants and their baseline characteristics have been presented before [2] and are shown in Figure S1 and Table S2. Forty-five participants finished the study. Due to technical problems, FMD measurements could not be performed for two participants on one test-day. For six participants, one measurement could not be interpreted. Therefore, FMD results are presented for 37 participants. Compliance to the treatments was confirmed by increases in fasting plasma total (aglycone and glucuronide conjugates) resveratrol and total dihydroresveratrol [2]. From the number of returned capsules, it was estimated that 99% (range: 88–111%) of the provided capsules were used on average. BMIs at the start and at the end of the placebo period were 28.4 ± 3.1 kg/m^2^ and 28.3 ± 3.1 kg/m^2^, respectively. For the *trans*-resveratrol period, these values were 28.3 ± 3.0 kg/m^2^ and 28.4 ± 3.1 kg/m^2^. Changes within or between treatment periods were not significantly different.

### 3.2. Vascular Function Measurements

Fasting FMD values at the end of the placebo and *trans*-resveratrol periods were not significantly different. In addition, changes in FMD after meal intakes were not different between both interventions (Table 1). Within the placebo group, we also did not observe an effect of the meal challenge on FMD. Comparing *trans*-resveratrol and the placebo, no differences were found between fasting arterial diameters or differences in postprandial changes. Furthermore, measures of arterial stiffness, PWV_cf_, AIx_p_, AIx_c_ and AIx_c_ adjusted for a heart rate of 75 beats per minute (AIx_c_HR75), were not affected by *trans*-resveratrol intake.

### 3.3. Postprandial Glycaemia and Lipaemia

**Figure 1:**
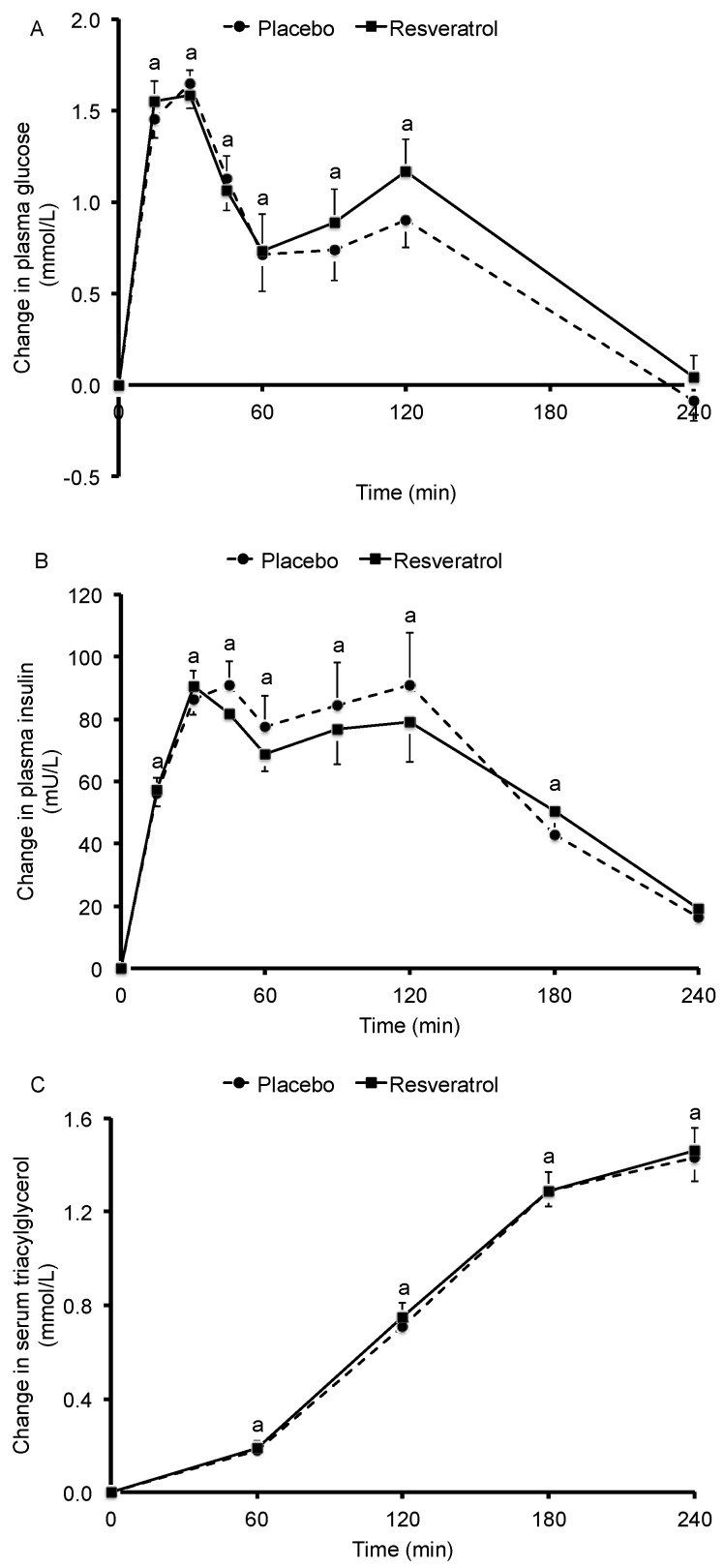
Baseline-corrected changes in plasma glucose (**A**), plasma insulin (**B**) and serum triacylglycerol (**C**) concentrations in 45 overweight and slightly obese men and women after consumption of two muffins and 300 mL of skim milk following a 4-week placebo period or a 4-week *trans*-resveratrol (150 mg daily) period. Values are means ± SEM. ^a^ Significant change from baseline; *p* < 0.05.

Fasting concentrations of plasma glucose, plasma insulin, and serum triacylglycerol did not differ between the test days at the end of the placebo and *trans*-resveratrol periods. After meal consumption, plasma glucose and plasma insulin concentrations significantly increased (*p* < 0.001 for time effect; Figure 1). These increases, however, were not significantly different between the two intervention periods. Serum triacylglycerol concentrations also increased significantly after the meal (*p* < 0.001 for time effect), but these effects were not changed by resveratrol supplementation. The iAUCs, AUCs and maximal changes in these parameters were also comparable between the two intervention periods (Table 2).

### 3.4. Postprandial Markers of Endothelial Activation and Inflammation

**Figure 2:**
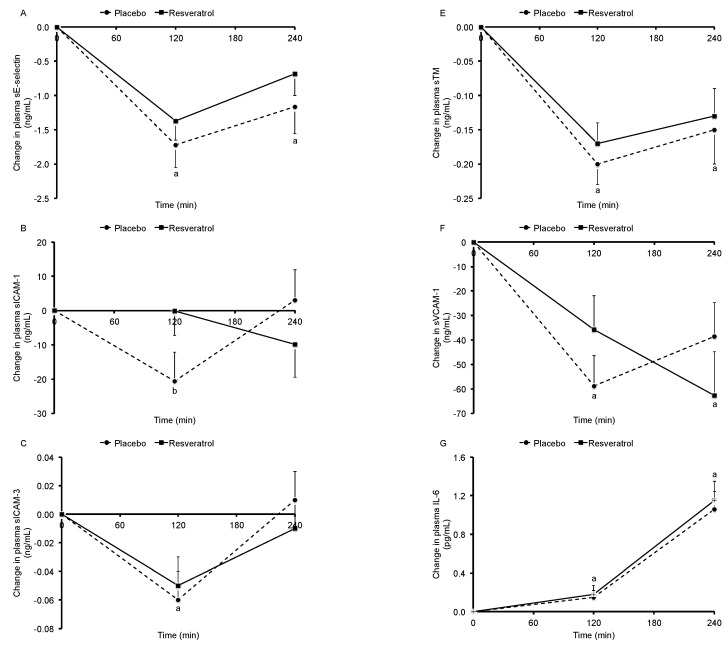

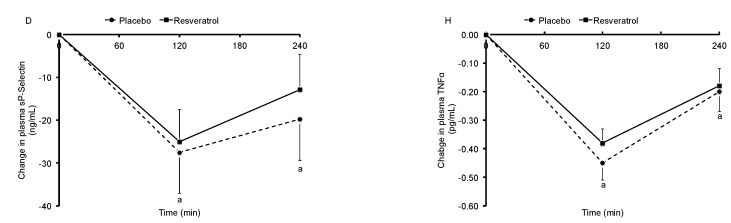
Baseline-corrected changes in plasma sE-selectin (**A**), sICAM-1 (**B**), sICAM-3 (**C**), sP-selectin (**D**), sTM (**E**), sVCAM-1 (**F**), IL-6 (**G**), and TNFα (**H**) concentrations in 45 overweight and slightly obese men and women after consumption of two muffins and 300 mL of skim milk following a 4-week placebo period or a 4-week *trans*-resveratrol (150 mg daily) period. Values are means ± SEM; sE-selectin: soluble endothelial selectin; sICAM-1: soluble intercellular adhesion molecule-1; sICAM-3: soluble intercellular adhesion molecule-3; sP-selectin: soluble platelet selectin; sTM: soluble thrombomodulin; sVCAM-1: soluble vascular cell adhesion molecule-1; IL-6: interleukin 6; TNFα: tumor necrosis factor alpha. ^a^ Significant change from baseline; *p* < 0.05. ^b^ Changes between the *trans*-resveratrol and placebo periods significantly different; *p* < 0.05.

Fasting concentrations of plasma markers for endothelial activation (sE-selectin, sICAM-1, sICAM-3, sP-selectin, sTM, sVCAM-1) and inflammation (IL-6, TNFα) were comparable between both intervention periods. No significant meal x time interactions were found, except for plasma sICAM-1 (*p* = 0.02). For T120, plasma sICAM-1 concentrations were higher after consuming *trans*-resveratrol (*p* = 0.04). For all other plasma markers, time effects were significant (*p* < 0.001; Figure 2).

### 3.5. Exploratory Data Analyses

Exploratory data analyses did not suggest that effects were dependent upon sex (25 men vs. 20 women) or degree of obesity (BMI ≥ 30 kg/m^2^; *n* = 16, 7 men and 9 women vs. BMI < 30 kg/m^2^; *n* = 29, 18 men and 11 women). When analyses were restricted to participants that did not use any prescribed medication (*n* = 31), conclusions were comparable as well.

## 4. Discussion

In this study on overweight and slightly obese men and women, a daily *trans*-resveratrol supplement of 150 mg for 4 weeks did not affect FMD, arterial stiffness plasma, or markers of endothelial function and low-grade inflammation in the fasting state or the postprandial phase.

In vitro and animal studies have suggested positive effects of resveratrol on endothelial function, possibly through effects on eNOS, NO bioavailability, SIRT1, and COX-1 [5,6,20,21]. However, in vivo resveratrol undergoes fast and extensive metabolic conversion, leading in humans to low plasma concentrations of resveratrol, but high concentrations of resveratrol metabolites, such as dihydroresveratrol monoglucuronide [22]. In the present study, we also found that fasting plasma dihydroresveratrol concentrations were about twice as high as those for resveratrol [2].

In humans, positive effects of *trans*-resveratrol supplementation on endothelial function have also been reported. In an acute study of 19 overweight and obese adults with elevated blood pressure, Wong et al. observed a dose-dependent effect of *trans*-resveratrol intake (0, 30, 90, 270 mg) on FMD [8]. Additionally, a daily supplement of 75 mg *trans*-resveratrol for 6 weeks increased FMD by 1.38 percent points from 5.83% (placebo) to 7.21% in 28 obese, but otherwise healthy adults (BMI 30–45 kg/m^2^) [9]. However, we found no effects on FMD of *trans*-resveratrol supplementation. In retrospect, the statistical power of our study was >95% to detect a true change in FMD of 1.38 percent points with an alpha of 0.05. Thus, our study was adequately powered to detect the effect observed by Wong et al. [9]. The reason for this discrepant finding is not clear. Our participants had a lower BMI, but exploratory subgroup analyses did not indicate an effect on FMD in the obese participants. It should be noted, however, that the statistical power for subgroup analyses might not have been sufficient. It is also possible that the duration of our study was not long enough, as the study by Wong et al. [9] lasted 6 weeks. Finally, FMD values in our study were lower than those measured by Wong et al. [9], although it is difficult to compare absolute FMD values between study sites due to different methodologies. It can be speculated that the endothelial function of our participants may have been too poor and was therefore not responsive to diet-induced changes within a 4-week period. On the other hand, it can be argued that a lower FMD would present more tendency to change or improve. Thus, an obvious reason to explain the difference in findings between our study and the study of Wong et al. is lacking [9]. A multicenter study using rigidly standardized measurements and addressing different aspects of endothelial function may be needed to increase the external validity of findings and to further clarify the effects of *trans*-resveratrol on the vasculature.

Participants also received a meal to assess the effect of 150 mg of *trans*-resveratrol on the postprandial FMD response. To disentangle the effects of long-term *trans*-resveratrol supplementation from those of an acute intake, we deliberately chose not to incorporate *trans*-resveratrol in the meal challenge. However, *trans*-resveratrol did not modify the postprandial change in FMD, even though the expected increase in serum triacylglycerol concentrations, which may deteriorate the postprandial FMD response, was observed [23]. Unexpectedly, FMD did not change at all during the postprandial period. Earlier, we have shown an impaired FMD 2 h after consuming a high-fat meal for overweight and slightly obese men [17]. The main difference between the two studies was the amount of protein in the test meal, which was lower in the latter study [17]. Indeed, a postprandial impairment in FMD of the brachial artery was not observed, when caseinate proteins were added to the mixed meal [24], which was explained by an increased supply of the amino acid L-arginine, a precursor for NO synthesis. Another explanation may be that in the present study, FMD was measured 4 h after meal intake, as opposed to 2 h in the earlier study. However, other studies did show a 4-hour postprandial deterioration in FMD after a high-fat meal [25,26]. As *trans*-resveratrol did not change postprandial plasma glucose, plasma insulin, or serum triacylglycerol concentrations, the absence of effects on FMD between the treatments was not confounded by differences in these blood parameters.

*Trans-*resveratrol supplementation did also not affect PWV_cf_ and pulse wave analyses in the fasting state or the postprandial phase. Wong et al. [9] also found no effects of *trans*-resveratrol on arterial compliance in their 6-week trial. To the best of our knowledge, no other studies assessed the effect of pure resveratrol on PWV_cf_. However, 4 weeks of pomegranate juice supplementation, a source of resveratrol, also had no effect on PWV_cf_ [27]. Dark chocolate, however, which is rich in flavanols, improved fasting PWV_cf_ within 4 weeks, while blood pressure did not change [28]. This suggests that the timeframe chosen in our study was not too short to demonstrate changes in PWV_cf_.

Anti-inflammatory effects of resveratrol have also been reported. In vitro, treating aortic endothelial cells treated with resveratrol at physiologically relevant concentrations inhibited TNFα induced NF-κB activity [29], reduced inflammatory gene expression [30], and decreased monocyte adhesiveness to the endothelium [31]. Additionally, when cultured human coronary artery endothelial cells were incubated with plasma from patients that had received 400 mg of *trans*-resveratrol for 4 weeks, inflammatory gene expression was inhibited [32]. In vivo, TNFα concentrations were lower after supplementation for 30 days with 150 mg of *trans*-resveratrol, although CRP, IL-1β, IL-6 and IL-8 concentrations were not affected [33]. Finally, a single oral dose of 100 mg of resveratrol and 75 mg of total polyphenols from a muscadine grape extract suppressed the mRNA and protein expression of inflammatory proteins induced by a high-fat, high-carbohydrate meal in healthy participants [34]. Effects, however, may have been due to the muscadine grape extract. In the present study, no effects were found on plasma concentrations of fasting or postprandial inflammatory and endothelial biomarkers. Only sICAM-1 concentrations increased 120 min after *trans*-resveratrol supplementation, compared with the placebo. After 240 min, concentrations were comparable. This effect was not observed for any of the other vascular function markers and may therefore have been due to chance, which warrants further study. In another study, sICAM-1 concentrations improved after 1-year consumption of a grape supplement including only 8 mg of resveratrol in patients who received statins in the primary prevention of cardiovascular disease [35]. Taken together, evidence that resveratrol supplementation changes in vivo plasma concentrations of endothelial and inflammatory biomarkers, is limited.

## 5. Conclusions

Vascular function as measured by FMD and PWV_cf_ did not change after 150 mg/day of *trans*-resveratrol supplementation for 4 weeks. Furthermore, the lack of effect on postprandial plasma glucose, plasma insulin, and serum triacylglycerol concentrations, as well as postprandial plasma endothelial and inflammatory biomarkers, extends our earlier findings [2] that 150 mg of *trans*-resveratrol daily for 4 weeks had no effects on these parameters in the fasting state.

## Figures and Tables

**Table 1:** Effects in overweight and slightly obese men and women of 4-week *trans*-resveratrol supplementation (150 mg daily) on FMD and arterial stiffness before and after a test meal.

	Placebo			Resveratrol			*p*_fasting_	*p*_change_
	Fasting	4 h after Meal Intake	Change after Meal Intake	Fasting	4 h after Meal Intake	Change after Meal Intake		
FMD (%)	3.0 ± 1.9	3.3 ± 2.5	0.2 ± 2.6	2.9 ± 1.4	2.2 ± 2	−0.7 ± 2.3	0.69	0.13
Baseline diameter (mm)	4.4 ± 0.8	4.3 ± 0.9	−0.1 ± 0.3	4.3 ± 0.8	4.3 ± 0.9	−0.1 ± 0.4	0.15	0.73
AIx_c_ (%)	32 ± 10	31 ± 7	−1 ± 7	32 ± 10	30 ± 9	−2 ± 6	0.79	0.59
AIx_c_HR75 (%)	24 ± 8	23 ± 8	−1 ± 5	25 ± 9	23 ± 8	−2 ± 6	0.46	0.62
AIx_p_ (%)	89 ± 13	86 ± 11	−3 ± 7	92 ± 17	89 ± 15	−3 ± 14	0.29	0.97
PWV_cf_ (*m*/*s*)	10.4 ± 2.7	10.9 ± 1.8	0.4 ± 2.6	11.0 ± 3.4	10.5 ± 3.1	−0.7 ± 2.7	0.16	0.09

Values are means ± SD; *n* = 45, except for FMD (*n* = 37), baseline diameter (*n* = 37) and PWV_cf_ (*n* = 40). FMD, flow-mediated vasodilation; AIx_c_, central augmentation index; AIx_c_HR75, central augmentation index adjusted for a heart rate of 75 beats per minute; AIx_p_, peripheral augmentation index; PWV_cf_, carotid-femoral pulse wave velocity. Fasting values (*p*_fasting_) and changes after meal intake (*p*_change_) during the *trans*-resveratrol period were compared with values obtained during the placebo period.

**Table 2:** Effects in overweight and slightly obese men and women of 4-week *trans*-resveratrol supplementation (150 mg daily) on postprandial glucose, insulin and triacylglycerol metabolism.

	Placebo	Resveratrol	*p*-Value
Plasma glucose			
iAUC (mmol·min/L)	187 ± 136	195 ± 147	0.59
AUC (mmol·min/L)	1421 ± 207	1421 ± 224	1.00
Maximal change (mmol/L)	2.08 ± 0.87	2.15 ± 0.79	0.48
Plasma insulin			
iAUC (U·min/L)	11.2 ± 11.0	10.5 ± 10.0	0.34
AUC (U·min/L)	13.5 ± 11.4	12.9 ± 10.4	0.34
Maximal change (mU/L)	131 ± 117	125 ± 95	0.43
Serum triacylglycerol			
iAUC (mmol·min/L)	169 ± 68	176 ± 74	0.32
AUC (mmol·min/L)	541 ± 203	566 ± 218	0.54
Maximal change (mmol/L)	1.49 ± 0.59	1.49 ± 0.62	0.94

Values are means ± SD, *n* = 45. iAUC, incremental area under the curve; AUC, area under the curve.

## Supplementary Materials

The following are available online at www.mdpi.com/2072-6643/9/6/596/s1: Figure S1: Participants flow chart; Table S1: Composition of the test meal; Table S2: Baseline characteristics of the participants.
