# Supplementary material for: Trans-Resveratrol Supplementation and Endothelial Function during the Fasting and Postprandial Phase: A Randomized Placebo-Controlled Trial in Overweight and Slightly Obese Participants
Source: Nutrients. 2017 Jun 12;9(6):596. doi: 10.3390/nu9060596 (PMC5490575; doi:10.3390/nu9060596)
Supplement: Supplementary file 1 [file nutrients-09-00596-s001.zip › Table S1.pdf]

Table S1. Composition of the test meal

Nutrient	2 muffins	300 mL Skim milk	Total
Energy, kJ	4095	503	4598
Energy, kcal	980	120	1100
Protein, g	14.5	12	26.5 (9.6 % of energy)
Carbohydrates, g	103	18	121 (44.0 % of energy)
Total fat, g	56.6	<0.1	56.6 (46.6 % of energy)
Saturated fatty acids, g	33.9	0	33.9 (27.7 % of energy)
Trans fatty acids, g	2.2	0	2.2(1.8 % of energy)
Monounsaturated fatty acids, g	14.5	0	14.5 (11.9 % of energy)
Polyunsaturated fatty acids, g	2.7	0	2.7 (2.2 % of energy)
Cholesterol, mg	349	0	349

The ingredients needed to prepare two muffins were butter (61.7 g), flour (60 g), sugar (60 g), one egg plus some vanilla sugar.
