# Supplementary material for: Trans-Resveratrol Supplementation and Endothelial Function during the Fasting and Postprandial Phase: A Randomized Placebo-Controlled Trial in Overweight and Slightly Obese Participants
Source: Nutrients. 2017 Jun 12;9(6):596. doi: 10.3390/nu9060596 (PMC5490575; doi:10.3390/nu9060596)
Supplement: Supplementary file 1 [file nutrients-09-00596-s001.zip › Table S2.pdf]

Table S2. Baseline characteristics of the participants

	Total			Men			Women		
Age, y	61	±	7	62	±	7	59	±	7
BMI, kg/m^2	28.3	±	3.2	28.5	±	2.9	29.2	±	3.6
Systolic BP, mmHg	136	±	17	138	±	16	135	±	18
Diastolic BP, mmHg	88	±	9	88	±	9	88	±	10
Heart rate, BPM	67	±	8	67	±	8	68	±	7
Plasma glucose, mmol/L	5.66	±	0.53	5.71	±	0.57	5.59	±	0.48
Serum total cholesterol, mmol/L	6.38	±	1.00	6.20	±	1.01	6.60	±	1.00
Serum HDL cholesterol, mmol/L	1.14	±	0.23	1.00	±	0.17	1.30	±	0.18

Values are means ± SDs, *n* = 45 (25 men and 20 women). BP, blood pressure; BPM, beats per minute
