# Supplementary figures and images for: Trans-Resveratrol Supplementation and Endothelial Function during the Fasting and Postprandial Phase: A Randomized Placebo-Controlled Trial in Overweight and Slightly Obese Participants
Source: Nutrients. 2017 Jun 12;9(6):596. doi: 10.3390/nu9060596 (PMC5490575; doi:10.3390/nu9060596)

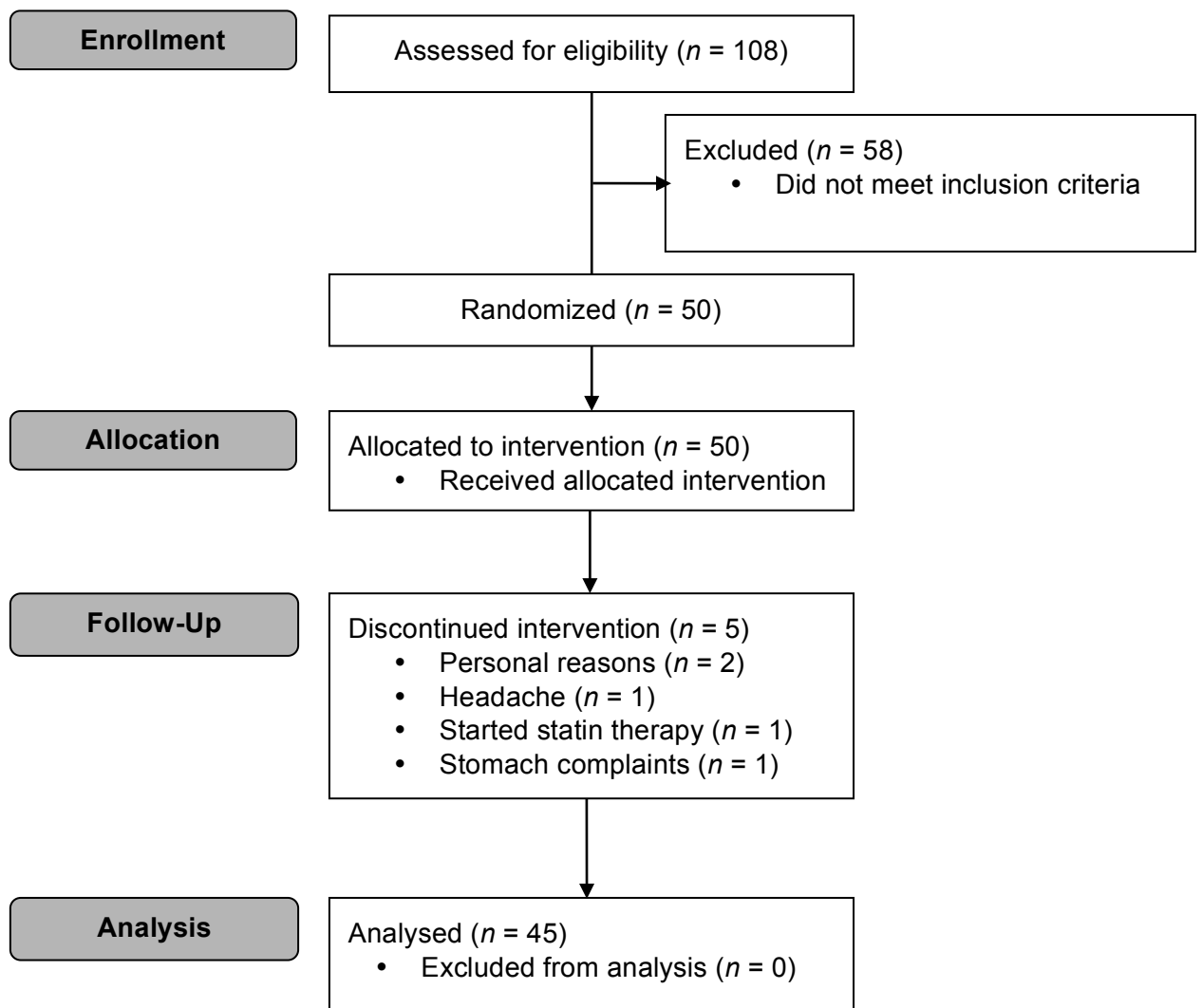

**Figure S1.** Participants flow chart

Supplement: Supplementary file 1 [file nutrients-09-00596-s001.zip › Figure S1.pdf]
